# Benign familial neonatal convulsions: A family with a rare disorder

**DOI:** 10.4103/0972-2327.40227

**Published:** 2008

**Authors:** Harbag Singh, Rajnish Raj

**Affiliations:** 1 Department of Neurology, Government Medical College, Rajindera Hospital, Patiala - 147 001, Punjab State, India; 2 Department of Psychiatry, Government Medical College, Rajindera Hospital, Patiala - 147 001, Punjab State, India

**Keywords:** Benign familial neonatal convulsions, computed tomography, electroencephalograph

## Abstract

The authors report a family from Punjab (India) with 10 members having benign familial neonatal convulsions (also known as benign familial neonatal seizures) in two generations. This disorder is quite rare. The clinical presentation of index case along with the findings of computed tomography of the brain and electroencephalograph is described. Important features of all the family members along with a brief review of the literature are also given.

## Synonym

Benign familial neonatal seizures.

## Introduction

Benign familial neonatal convulsions (BFNC, also known as benign familial neonatal seizures) constitute a rare, dominantly inherited disorder characterized by frequent brief seizures within the first days of life.[1] Seizures are clonic, brief, and recur frequently for up to 7 days. Although other seizure types may develop later in infancy or childhood, various aspects of development are unaffected. Brief seizures occur for several days or weeks and then regress spontaneously. There is a positive family history with an autosomal dominant inheritance pattern for neonatal seizures. Affected individuals show normal psychomotor development. Electroencephalograph (EEG) changes are not specific to the condition. Interictal EEG may be normal or mildly/moderately abnormal with focal or multifocal abnormalities. Ictal EEG starts with a brief flattening followed by asymmetric spike and wave complexes of 1-2 min duration.[2]

It was the first idiopathic epilepsy in which linkage analysis was successful. The gene for the first locus (EBN_1_), KCNQ_2_, was identified by characterization of a submicroscopic deletion on chromosome 20q13.3 and it shows significant homology with a voltage-dependent delayed rectifying potassium channel gene, KCNQ1.[3] Another gene, KCNQ_3_, was mapped to the second locus (EBN_2_) on chromosome 8q24 and was found to be mutated in affected members of a BFNC/EBN2 family.[4]

We report a family affected by this rare disorder in which 10 members were affected in two generations.

## Case

An 8-month-old female child reported with history of seizures since the age of 4 days. She was born to a primigravida, at full term with normal vaginal delivery. There was nothing significant in the ante-partum history. The child looked well and active. Developmental milestones were normal. Head circumference was 42.3 cm (within normal limits). Fontanellae were normally palpable. There were no craniofacial abnormality, organomegaly or neurocutaneous markers.

Computed tomography (CT) scan (head) was normal. EEG graph included normal drowsy and sleep-state patterns, which were consistent with the age of the patient. Background EEG frequency ranged between 2 and 5 Hz with amplitude of 150-200 μV. At places, sleep markers in the form of V-waves, K-complexes, and sleep spindles were also seen. There was no epileptiform activity.

Seizure description included initial tonic motor activity, which was followed by asymmetrical facial movements and clonic activity. These were accompanied by uprolling of eye balls and unconsciousness, and the child used to get cyanosed during these episodes. The parents reported that the total duration of the seizure was between 5 and 15 min. Frequency of seizures varied from 3 to 4 per week to 1 per month. Parents were reassured regarding the benign nature and excellent prognosis in this disorder.

**Figure 1:**
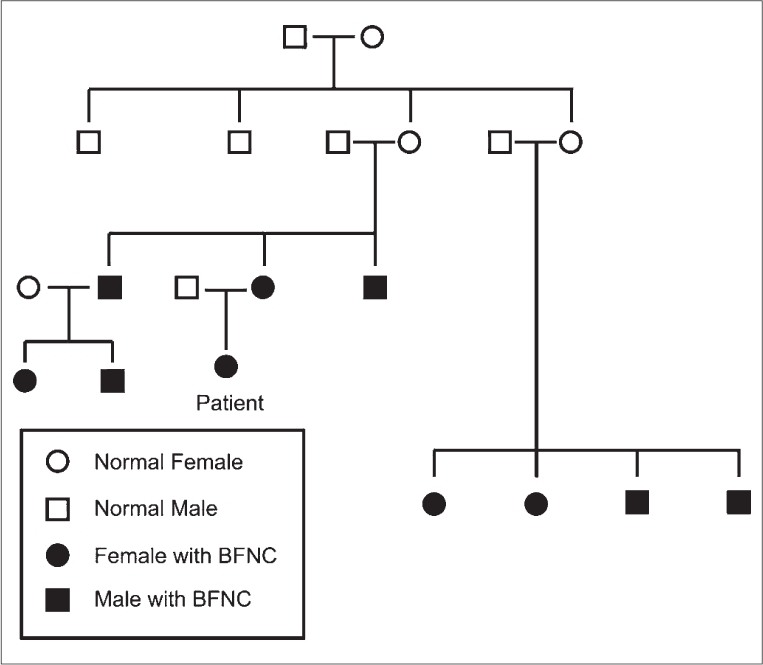
Pedigree of a family with Benign Familial Neonatal Convulsion involving 10 members in two successive generations

There was very strong family history of seizures involving nine cases in two successive generations [Figure 1]. Males and females were almost equally involved. The age of onset was remarkably constant. The seizure phenomenology and its occurrence on the 4^th^ day of life were similar among all these cases and it resembled the description as noted above. The age of remission was also similar in five out of nine cases. In these cases, the remission occurred at the age of 1¼ months. In other two cases, remission occurred at the age of 1 year. One patient had remission at the age of 1¼ years. Another patient behaved differently, while he had initial remission at the age of 1¼ years, seizures recurred after 3 months though with different ictal manifestations. Presently, he is 22 years old and is continuing to get seizures. All the patients described above were neurologically normal. The index case presently 2½ years old also got remission of seizures at the age of 1 year and is having normal developmental milestones [Table 1].

**Table 1:** Seizures onset and remission

Age (yrs)	Sex	Age of onset	Course
32	M	4^th^ day	Remission at 1¼ months
29	F	4^th^ day	Remission at 1 year
28	F	4^th^ day	Remission 1¼ months
26	F	4^th^ day	Remission at 1¼ months
25	M	4^th^ day	Remission at 1¼ months
24	M	4^th^ day	Remission at 1¼ months
22	M	4^th^ day	Initially had remission at 1¼ years age but remission lasted only 3 months. The semiology of the later seizures differed from the earlier ones.
5½	F	4^th^ day	Remission at 1¼ years
3½	M	4^th^ day	Remission at 1 year
2½	F	4^th^ day	Remission at 1 year

## Discussion

This is quite a rare disorder. Following its first report in 1964, another report appeared in 1968 describing 14 family members in five generations.[5] In 1979, another family with 15 affected members was reported.[6] Thereafter, similar families were reported in 1982,[7] with patterns of inheritance compatible with an autosomal dominant trait. A review comprising of 14 families with 87 individuals having benign familial neonatal convulsions (BFNC) was published in 1985.[8] Up to 1997, there were reports of only 44 families with 355 affected members.[9] Probably only one family with this disorder has been reported from India.[10] The rarity of this disorder may be artifactual, given the benign nature of this disorder. Families, possibly from their experiences, can predict its benign nature. They rarely seek medical help unless the seizures do not remit by the usual age. Self-limiting disorders become socially acceptable and many a times are ascribed to supernatural powers in developing countries.

This family represents a “prototypic” description of this disorder. All the members had seizures on 4^th^ day of life. Onset is commonly in the 1^st^ week of life, but in one-third of the cases, it may be in the first 1-2 months.[1] Some authorities have stressed the onset on 2^nd^ or 3^rd^ day of life.[11] However, narrowing down the onset to specific days of life seems unwarranted, because of variations as exemplified by the family described here. The variation is probably determined developmentally because infants born prematurely will have seizure onset at an older chronological age than the infants born at term.[12] Seizure phenomenon starting with tonic motor activity, progressing to clonic movements with asymmetric involvement of face, repetitive contraction of orbicularis oculi muscle, uprolling of eye balls, and unconsciousness resembles the clinical description in other reports. Cyanosis in this case is likely to be due to autonomic phenomenon. Cyanosis can also occur in case of prolongation of tonic phase of seizures, which, however, is not usual in this disorder. Parents have described the duration of seizures to be 5-15 min. In the published literature, seizures are described as brief (1-2 min). There is likely to be an error in recording, as the parents usually cannot note duration of seizures because of panic and nervousness at such times. The seizure frequency (once in 2 days to once in 1 month) is considerably less in 20-30 per day as reported in the earlier literature.[1] As reported previously, boys and girls are equally affected in this family.[1] This denotes that the disorder has autosomal inheritance. Normal neuroimaging and interictal EEG also follow the usual pattern.

The prognosis in this family matches the description in previous reports, e.g., remission by 6 weeks in approximately 2/3^rd^ of cases and development of afebrile seizures in 10-14%.[1] The psychomotor development, predictably, is normal among all the members of this family. However, 5 out of 10 members crossed the upper age limit of normal age for remission, i.e., 6 months. This report emphasizes the importance of family history in the diagnosis of this disorder. Diagnosis of this disorder can avoid needless investigations and toxic medications.
